# Lifestyle behaviors trend and their relationship with fear level of COVID-19: Cross-sectional study in Saudi Arabia

**DOI:** 10.1371/journal.pone.0257904

**Published:** 2021-10-13

**Authors:** Shaima A. Alothman, Abdullah F. Alghannam, Alaa A. Almasud, Arwa S. Altalhi, Hazzaa M. Al-Hazzaa

**Affiliations:** Lifestyle and Health Research Center, Health Science Research Center, Princess Nourah bint Abdulrahman University, Riyadh, Saudi Arabia; University of Maiduguri College of Medical Sciences, NIGERIA

## Abstract

**Introduction:**

COVID-19 pandemic cautionary measures have affected the daily life of people around the globe. Further, understanding the complete lifestyle behaviors profile can help healthcare providers in designing effective interventions and assessing overall health impact on risk of disease development. Thus, this study aims to assess the complete spectrum of lifestyle behaviors (physical activity, sedentary behavior, sleep, distress, social support, dietary habits, and smoking) prevalence and its association with fear of COVID-19 in people living in Saudi Arabia.

**Methods:**

Self-administered survey consisted of seven sections was used to collect data on fear of COVID-19 using Fear of COVID-19 Scale (FCV-19S), physical activity and sedentary behavior using the Global Physical Activity Questionnaire (GPAQ), sleep quality using the Pittsburgh Sleep Quality Index (PSQI), psychosocial distress using Kessler Psychological Distress Scale (K-10), social support using the MOS social support survey, and dietary habits using a short version of food frequency questionnaire (FFQ). The online survey was distributed via social media platforms during lockdown period of COVID-19 pandemic (May–June 2020). Each section consisted of validated questionnaire examining one of aforementioned lifestyle behaviors. Associations were analyzed using multiple linear regression.

**Results:**

A total of 669 individuals attempted to complete the online survey, 554 participants completed at least 2 sections of the survey (82.8%), and 41.3% (n = 276) completed the whole online survey. The majority of the sample were female (83%), not smokers (86.5%), had sufficient sleep duration (7.5 hrs ± 2.1), and only indicated mild level of distress (21.4 ± 8.9); they also reported high level of sedentary behavior (7.7 hrs ± 4.5), poor sleep quality (5.4 ± 2.4), were not engaged in healthy eating habits, and moderate level of perceived social support (62.0% ± 27). Only physical activity results indicated that about half of the sample were engaged in moderate to vigorous level of physical activity (54.3%). Further, being female (β = 0.12; 95% CI: 0.45, 2.94) and married (β = 0.13; 95% CI: 0.3, 2.63) were associated with fear of COVID-19 level (β = 0.21; 95% IC: 0.05, 0.19) with a confidence interval level of 95%. In addition, distress was associated with fear.

**Conclusion:**

The trend of lifestyle behaviors measured during lockdown period changed from previously published rates. Future research needs to establish the short-term and long-term effect of lifestyle behaviors complete profile on physical and mental health.

## Introduction

SARS-CoV-2 (COVID-19) affects mainly the respiratory system with potential dire health consequences [1, 2]. The high health burden and infectious rate of the COVID-19 along with the absence of a readily available vaccines or treatment prompted the world health organization to declare COIVD-19 as a global pandemic on March, 2020 [3]. This has led nations around the world to enforce a series of cautionary and emergency procedures such as school closure or nation-wide curfews [4]. In Saudi Arabia, these procedures included switching to distance learning, working from home to all non-medical or security personnel, nation-wide curfews, and some city lockdown. In addition to these public health measures, people were requested to practice social distancing, which is described as maintaining a distance of 2 m between yourself and other people and avoiding social gatherings, limiting contact with older individuals and those in poor health, avoiding common greetings such as handshakes, and avoiding crowded places and non-essential gatherings [5]. All these procedures are essential to slow the spread of the virus and lessen the burden on healthcare system [6, 7].

While these cautionary and emergency procedure measures were necessary to lessen the burden of COVID-19 on the health system and decrease the number of fatalities associated with the disease, the same cautionary measures have also affected the daily life of most Saudis. These effects might include increased fear level of the disease itself and changes in lifestyle behaviors. The high infectious rate and mortality of COVID-19 might have increased fear level of COVID-19 worldwide [8, 9]. Fear of infectious diseases is directly linked to fear of transmission and the disease associated mortality and morbidity; Further, this usually leads to increase levels of stigmatization, discrimination, and loss [9, 10]. Further, it may lead to a decrement in healthy lifestyle behaviors and poor psychological well-being, however, this is yet to be investigated examined in Saudi Arabia. Altogether, the association between lifestyle behaviors and fear of COIVD-19 remains unclear.

Whether acute or prolonged, the negative changes in lifestyle behaviors such as lower level of physical activity, high level of sedentary behavior, poor sleep, or poor psychological well-being may result in a surge in incidence of comorbidities [10, 11, 12]. Research shows that health-related lifestyle behaviors such high levels of physical inactivity, sedentary behavior, and distress are associated with increased risk of developing chronic diseases and increased mortality rate [13–15]. Further, studies that examined the whole spectrum of lifestyle behaviors showed that indeed negative lifestyle behaviors tend to cluster [16–18]. These studies were conducted in populations with different cultures and moral values than those in the Middle East, therefore, their results cannot be generalized globally without further assessment [19].

Furthermore, the current COVID-19 pandemic cautionary and emergency procedures might heighten the negative impact of unhealthy lifestyle behaviors. For example, an Australian study showed that poor psychological health was associated with negative changes in lifestyle behaviors such as physical activity, sleep, smoking and alcohol intake [20]. Another study conducted in Italy showed that level of physical activity decreased significantly during social distancing time, and this was associated with worst psychological well-being [21]. while a Canadian study showed that poor psychological well-being was associated only with physically inactive people [22]. All of this highlight the importance to instill healthy lifestyle behaviors habits such as regular exercise or moving throughout the day to avoid negative impact on health in crisis times [23, 24].

Understanding the complete lifestyle behaviors profile could aid healthcare providers in designing effective interventions and assessing overall health impact on risk of disease development. Thus, assessment of lifestyle behaviors during the period of cautionary and emergency procedures is needed to anticipate and prepare to prevent any lasting health and social impact of COIVD-19 on the population. In accordance, the present study aims to investigate the level of lifestyle behaviors (physical activity, sedentary behavior, sleep, social distress, social support, dietary habits, and smoking) in people living in area with stay home advisory implemented in Saudi Arabia. Further, the study aimed to assess the relationship between fear level of COIVD-19 and lifestyle behaviors.

## Material and methods

### Study design

A cross-sectional study was conducted on all individuals who live in a region with stay home advisory implemented. Inclusion criteria were individuals aged 18 and older and living in Saudi Arabia at the time completing the survey. Participants who had been diagnosed with COVID-19 or suspected COVID-19 diagnosis were excluded from the study.

### Participants and recruitment

Data collection was based on the distribution of an electronic survey through social media and electronic media platforms including “WhatsApp” and “Twitter”. The distribution of an electronic survey through WhatsApp” and “Twitter” was initiated from lifestyle and Health Research Center employee’s phones. The survey was distributed electronically to all participants including all nationalities who live in different regions of Saudi Arabia. The study used a snowball sampling technique where individuals received the study invitation link were asked to forward the link of the study regardless of their participation in the study survey or not.

The sample size was calculated to be 377 Participants (using the following sample size equation for proportions: n = (z^2^pq)/d^2^), while assuming a population proportion that yields the maximum possible sample size required (p = 0.50), with a confidence level of 95% and a margin of error of 5%. However, because this study aims to recruit participants form different age groups, regional areas, and gender the sample size calculation all responses to the survey was included in the final analysis.

Interested potential participants recruited via electronic media platforms click on the study invitation link then they were directed to online welcoming. On this page participants found the study aims, and survey instructions were described. An electronic informed consent was filled by participants before participating and answering the survey’s questions. Participants were informed that they are voluntarily participating and may drop out at any time or not answer any of the survey questions. Participants were assured of the confidentiality of information. Participants had to answer a question if they had been diagnosed of COVID-19. The research procedure was conducted according to the principles expressed in the Declaration of Helsinki. The study was approved by the Institutional Review Board (IRB number 20–0142) at the Health Sciences Research Center at Princess Nourah Bint Abdulrahman University.

Participants completed the online survey using an online survey software (Redcap) [25, 26] between May and June 2020. Participants who did not complete an individual questionnaire were excluded for this specific questionnaire.

### Measures

Self-administered survey consisted of seven sections was used to collect data on demographics, fear of COVID-19, physical activity, sedentary behavior, sleep quality, psychosocial distress, social support, and dietary habits.

### Anthropometric measurements and demographic variables

Participants were asked to self-report their anthropometric measurements and demographic information, due to lockdown constraints prohibiting physical measurements. Anthropometric measurements included height, body weight to calculate body mass index (BMI). Demographic variables reported were age, city and region, marital status, smoking status, educational level, presence of chronic diseases, and employment type.

### Fear of COVID-19

Fear of COVID-19 was measured using Fear of COVID-19 Scale (FCV-19S). The scale consists of seven items pertaining to emotional fear reactions towards the pandemic. The original and translated Arabic version has shown to be reliable and valid [27, 28]. Participants were requested to respond on a five-item Likert-type scale ranging from 1 (*“strongly disagree”*) to 5 (“*strongly agree”*). The total score ranges between 7 and 35, with a higher sum score indicating a higher fear of COVID-19.

### Physical activity and sedentary behavior

Sedentary behaviors and physical activity of participants were self-reported by completing the Global Physical Activity Questionnaire (GPAQ) [29, 30]. GPAQ-A is a modified version of the International Physical Activity Questionnaire developed by the WHO. The Arabic version of the questionnaire had been tested for its validity and reliability [31, 32]. The questionnaire consists of 16 questions in three main domains to assess physical activity include activity at work, transport-related, and recreational activities. It collects information on participant physical activity frequency (time/week), duration (min), and intensity (light, moderate, and vigorous) during a typical day [30]. Physical activity level was categorized to high, moderate, and low. High physical activity level indicate that the participant was engaged in vigorous intensity activity on at least 3 days achieving a minimum total physical activity of at least 1500 MET minutes a week, or seven or more days of any combination of walking, moderate intensity or vigorous intensity activities achieving a minimum total physical activity of at least 3000 MET minutes a week. Where, moderate physical activity indicates the participant was engaged in five or more days of any combination of walking, moderate intensity or vigorous intensity activities achieving a minimum total physical activity of at least 600 MET minutes a week. Finally, low physical was given to participant if level of physical activity does not reach the criteria for either high or moderate levels of physical activity).

Sedentary behavior was classified by answering a question about the duration of sitting or reclining time at work, at home, traveling in car, bus, train, reading, and watching television, but do not include time spent sleeping on a typical day [32].

### Sleep quality

Sleep quality was assessed by completing the Pittsburgh Sleep Quality Index (PSQI) over a 1-month time interval [33]. The measure consists of 19 individual items, creating seven components that produce one global score ranges from 0 to 21. Poor sleepers have ≥ 5 scores as a cutoff global score. The Arabic version of PSQI has been shown to be valid and reliable [34].

### Psychosocial factors

The presence of psychological distress factors was determined by use of Kessler Psychological Distress Scale (K-10) [35]. In this study, the Arabic version of K10 scale was used which has been validated [36]. It comprises 10 items that were answered using a five-point scale to assess the level and severity of distress. It intended to yield a global measure of distress based on questions about anxiety and depressive symptoms that the participants had experienced in the most recent 4-week period. Participant responded to each question by rating from 1 (none of the time) to 5 (all of the time). Total scores vary from 10 to 50, with higher scores indicating high level of psychological distress (mild 20–24, moderate 25–29, severe 30 and above) [36].

### Social support

Perceived social support was evaluated with the MOS social support survey. It assessed social support within five domains: Emotional/ informational support, tangible support, affectionate support, and positive social interaction [37]. The original and translated Arabic version has shown to be reliable and valid [38]. It consists of 19 items answered by means of 5-point Likert type scale with responses options ranging from 1 ("never"); 2 ("seldom"); 3 ("sometimes"); 4 ("almost always") and 5 ("always") [37]. A sum of all item scores is calculated to create a total score ranging from 19 to 95. Total scores were then transformed to percentage following formula: [(observed total score– 19 / 95–19) * 100]. Higher percentage suggest a greater perception of social support.

### Dietary habits

Dietary intake was assessed using a short version of food frequency questionnaire (FFQ) over a specified period of time aiming to capture individual-level dietary intake (data not reported) [39]. The Arabic version of the questionnaire has been tested for its validity and reliability on Saudi population [40]. Food frequency questionnaire includes multiple-choices question about meals and light snacks consumption which participant self-reported the preferred meal or snack. Number of meals per day was used to assess dietary habits in this study.

### Data and statistical analyses

Data were checked, cleaned, and analyzed using SPSS-25 software (SPSS Inc., Chicago, IL, USA). Descriptive statistics for sociodemographic, lifestyle behaviors, and lifestyle behaviors divided by sex were summarized using frequencies for categorical variables or means and standard deviations (SD) for continuous variables. The normality was tested for all variables, non-normally distributed data were reported as median and inter-quartile rage. Chi-square or students *t*–test was used to compare sociodemographic data between participants completed at least 2 sections of the survey and those only completed the sociodemographic section.

Multiple linear regression models were used to examine the relationship between fear of COVID-19 and lifestyle behaviors with sociodemographic (age, sex, BMI, marital status, level of education, working status, geographic region, and number of comorbidities). Further, unadjusted and adjusted models of multiple linear regression were used to assess the association between fear of COVID-19 and lifestyle factors (smoking status, PA, SB, sleep, dietary habits, distress, and social support). Univariate analyses were conducted for all independent variables and any variable with p-value ≤ 0.1 was included in the adjusted models. The normality and homogeneity of the residual was tested during model development. All independent variables were tested for multicollinearity via Variance Inflation Factors (VIF); a score of ≥ 5 was considered indictive of multicollinearity. An alpha level of 0.05 assessed the significance of all relationships.

## Results

A total of 669 individuals attempted to complete the online survey, from which 82.81% (n = 554) completed at least 2 sections of the survey and 41.26% (n = 276) completed the whole online survey. A comparison of a sociodemographic data of participants who completed at least 2 sections of the survey (completed at least 2 survey sections group) and participants who only completed the first section (only demographics section group) indicated that only demographics section group seems to be older, married or divorced, have an undergraduate degree or less, do not currently work, and have more than one comorbidity Table 1.

**Table 1:** Sociodemographic data comparison between people who completed 2 survey sections with people who only completed the demographics section.

Variable		Completed at least 2 survey sections (n = 554)	Only demographics section (n = 115)	P-value
Age^1^		34.50 ± 11.68	40.77 ± 13.15	<0.00*
BMI^1^		25.09 ± 8.76	27.24 ± 5.73	0.22
Sex	Female	82.86 (459)	80.53 (91)	0.78
	Male	17.15 (95)	19.47 (22)	
Marital status	Single	39.53 (219)	18.75 (21)	<0.00*
	Married or divorce	60.47 (335)	81.25 (91)	
Geographic region	Central	65.34 (362)	75.68 (84)	0.20
	North	7.04 (39)	6.31 (7)	
	South	2.71 (15)	3.60 (4)	
	East	8.30 (46)	4.50 (5)	
	West	16.61 (92)	9.91 (11)	
Education level	Undergraduate degree or less	77.8 (431)	85.32 (93)	<0.00*
	Post graduate degree	22.2 (123)	14.68 (16)	
Working status	Do not work	34.3 (190)	56.44 (57)	<0.00*
	Onsite	16.79 (93)	18.81 (19)	
	Online	38.90 (216)	13.86 (14)	
	On vacation	9.93 (55)	10.89 (11)	
Number of comorbidities	0	71.66 (397)	57.39 (66)	<0.00*
	1	20.04 (111)	16.52 (19)	
	>1	8.30 (46)	26.09 (30)	

Data compared using chi-square test except for age and BMI where data were compared using student’s *t*-test^1^. Data reported as mean ± SD or Frequency (n).

*Significant at *p*<0.05

The overall results showed that participants had low level of fear related to COVID-19, were mostly non-smoker, indicated mild level of distress, and moderate levels of social support Table 2. In terms of physical activity only 9% engaged in high levels of physical activity, whereas half of the remaining sample reported moderate levels of physical activity. Further, majority of participants reported sitting for an average of six hours per day and did not consume three regular meals per day. Lastly, participants reported sleeping for seven hours in average per day with majority of them rate their sleep quality as poor. In Table 3, the results indicated that female participants expressed higher level of fear and distress compared to male participants.

**Table 2:** Level of fear of COVID-19 and lifestyle behaviors (sedentary behavior, physical activity, dietary habits, social relationships, distress, and sleep duration and quality).

Variable		mean ± SD or Frequency (n)
Fear of COVD-19 scale		16.28 ± 5.49
n = 554		
Smoking status	Yes	9.75 (54)
n = 554		
	No	86.46 (479)
	Started smoking with COVID-19	0.36 (2)
	Quit	1.62 (9)
	Do not want to disclose	1.81 (10)
Physical activity	Low	45.71 (208)
n = 455		
	Moderate	45.27 (206)
	High	9.01 (41)
Sedentary behavior (Hours)	Mean ± SD	7.67 ± 4.51
n = 440		
	Median (IQR)	6 (4–10)
	% of ≥ 6 hours	60.32 (265)
Eating habits (number of meals per day)	0	5.14 (19)
	1	20.00 (74)
n = 370		
	2	45.95 (170)
	3	28.92 (107)
Sleep	Duration (Hours)	7.52 ± 2.08
n = 318		
	% slept for 7–9 hours	55.03 (175)
	Quality (PSQI score)	5.36 ± 2.38
	% Good Sleepers	42.45 (135)
Distress	Mean ± SD	21.38 ± 8.95
n = 276	Mild %	11.23 (31)
	Moderate %	16.30 (45)
	Severe %	18.84 (52)
Social support %	Mean ± SD	62.00 ± 27
n = 276		
	% of Good support	28.62 (79)
	% of Moderate support	30.07 (83)
	% of No to Mild support	41.30 (114)

Data reported as mean ± SD or Frequency (n)

* Significant P-value set at < 0.05.

**Table 3:** Level of fear of COVID-19 and lifestyle behaviors divided by sex in the general public living in areas with stay home advisory implemented in Saudi Arabia.

Variables		Female	Male	P value
Fear of COVD-19 scale		16.48 ± 5.57	15.16 ± 4.97	0.03*
		n = 459	n = 95	
n = 554				
Smoking status^1^	Yes	6.10 (28)	27.36 (26)	<0.00
n = 554				
	No	91.07 (418)	64.21 (61)	
	Started smoking with COVID-19	0 (0)	2.11 (2)	
	Quit	1.09 (5)	4.2 (4)	
	Do not want to disclose	1.74 (8)	2.11 (2)	
Physical activity^1^	Low	45.11 (171)	48.68 (37)	0.45
n = 455				
	Moderate	45.11 (171)	46.05 (35)	
	High	9.76 (37)	5.26 (4)	
Sedentary behavior (Hours)		7.50 ± 4.55	8.59 ± 4.21	0.06
		n = 364	n = 76	
n = 440				
Eating habits (number of meals per day)^1^	0	4.22 (13)	9.68 (6)	0.10
	1	21.42 (66)	12.90 (8)	
	2	46.75 (144)	41.94 (26)	
n = 370				
	3	27.60 (85)	35.48 (22)	
Sleep	Duration (Hours)	7.60 ± 2.02	7.18 ± 2.32	0.18
		n = 263	n = 55	
n = 318				
	Quality (PSQI score)	5.24 ± 2.41	5.89 ± 2.18	0.07
		n = 263	n = 55	
Distress		21.90 ± 8.80	18.98 ± 9.34	0.04*
n = 276				
		n = 227	n = 49	
Social support		66.69 ± 20.14	65.59 ± 21.68	0.73
n = 276		n = 227	n = 49	

Data compared using student’s t-test or chi-square^1^; Data reported as mean ± SD or Frequency (n)

* Significant P-value set at < 0.05; PSQI, Pittsburgh sleep quality index.

The normality and homogeneity of the residual assumption was met for both models. Further, VIF test for multicollinearity indicated no concerns for multicollinearity in tested variables. A multiple linear regression analyses was performed to characterize the relationships between fear of COVID-19 and lifestyle behaviors with sociodemographic variables: age, sex, BMI, marital status, Education level, working status, geographic region, and number of comorbidities Table 4. Fear of COVID-19 showed a significant association with sex (β = 0.12; 95% CI: 0.45, 2.94) and marital status (β = 0.13; 95% CI: 0.3, 2.63). Further, sedentary behavior was associated with sex and marital status while age was associated with age and distress was associated with sex and number of cormorbidies. A second multiple regression linear analysis was performed to characterize the relationship between fear of COVID-19 and lifestyle behaviors: sedentary behavior, physical activity, dietary habits, social relationships, distress, and sleep duration and quality Table 5. Fear of COVID-19 showed a significant association with distress (β = 0.21; 95% CI: 0.05, 0.19). This association remained in the adjusted model.

**Table 4:** The relationship between fear of COVID-19 (n = 276) and lifestyle behaviors with sociodemographic variables (age, sex, BMI, marital status, education level, working status, geographic region, and number of comorbidities).

	Fear of COVID-19	Smoking status	Physical activity	Sedentary behavior	Eating habits	Sleep—Duration	Sleep—Quality	Distress	Social support
Variables	B (SE)	B (SE)	B (SE)	B (SE)	B (SE)	B (SE)	B (SE)	B (SE)	B (SE)
Age	0.02 (0.03)	0.00 (0.00)	-0.01 (0.00)	-0.03 (0.02)	0.00 (0.01)	- 0.4 (0.01)*	0.02 (0.02)	-0.20 (0.07)**	-0.04 (0.16)
Sex	-1.71 (0.63)**	-0.12 (0.07)	-0.08 (0.08)	1.41 (0.57)*	0.02 (0.12)	-0.05 (0.32)	0.47 (0.37)	-1.53 (1.44)	-1.56 (3.42)
Marital status	1.48 (0.60)*	-0.02 (0.06)	0.02 (0.08)	-1.76 (0.54)**	-0.06 (0.11)	0.05 (0.92)	-0.26 (0.34)	-1.49 (1.33)	4.23 (3.18)
Working status	0.36 (0.23)	-0.04 (0.02)	0.4 (0.03)	0.35 (0.21)	-0.01 (0.04)	0.03 (0.11)	0.08 (0.13)	-0.22 (0.51)	-0.78 (1.22)
Geographic region	0.26 (0.49)	-0.01 (0.05)	0.00 (0.06)	-0.29 (0.44)	0.03 (0.09)	-0.30 (0.23)	0.46 (0.29)	-0.34 (1.12)	2.34 (2.66)
Educational level	-0.87 (0.58)	0.11 (0.06)	0.01 (0.07)	-0.30 (0.51)	0.18 (0.11)	-0.27 (0.27)	0.40 (0.32)	-1.16 (1.24)	-2.13 (2.94)
BMI	-0.01 (0.03)	-0.00 (0.00)	0.01 (0.00)	0.03 (0.03)	0.01 (0.01)	-0.00 (0.01)	-0.00 (0.02)	0.13 (0.07)	-0.10 (0.17)
Number of Comorbidities	0.33 (0.32)	0.03 (0.03)	0.04 (0.04)	0.35 (0.29)	-0.02 (0.06)	0.01 (0.16)	0.21 (0.19)	1.70 (0.77)*	2.09 (1.82)
R^2^	0.04	0.02	0.02	0.07	0.02	0.06	0.04	0.11	0.02
F statistic	2.81 *	1.16	0.92	4.28**	0.97	2.60*	1.57	3.96**	0.76

*Significant at p<0.05

** Significant at p<0.01, SE: standard error.

**Table 5:** The relationship between fear of COVID-19 (n = 276) and lifestyle behaviors (smoking status, PA, SB, sleep, dietary habits, distress, and social support).

	Unadjusted model	Adjusted model^!^
Variables	B (SE)	B (SE)
Smoking status	-0.26 (0.48)	-0.22 (0.48)
Physical activity level	0.53 (0.50)	0.61 (0.50)
Sedentary behavior	-0.11 (0.07)	-0.07 (0.08)
Sleep	0.02 (0.15)	0.06 (0.16)
Dietary habits	0.02 (0.38)	0.00 (0.38)
Distress	0.12 (0.04)*	0.14 (0.4)*
Social support	-0.02 (0.02)	-0.02 (0.02)
R^2^	0.08	0.09
F statistic	3.15*	2.43*

*Significant at p<0.05, SE: standard error.

^!^Adjusted model for age, sex, marital status, and number of comorbidities.

## Discussion

Lifestyle behaviors are important indicators of health. Thus, the objective of this study is to assess the current level of lifestyle behaviors during COVID-19 cautionary and emergency procedures. A secondary objective was to investigate the relationship between fear of COVID-19 level and lifestyle behaviors. The study results indicated a divergence between good and poor lifestyle behaviors. The majority of the sample were non-smokers, had sufficient sleep duration, and only indicated mild level of distress. Conversely, they also reported high level of sedentary behavior, poor sleep quality, were not engaged in healthy eating habits, and moderate level of perceived social support. Only physical activity results indicated that the half of the sample were engaged in moderate to vigorous physical activity (MVPA) level of physical activity.

Prevalence of MVPA level in this study were much higher than previously reported national prevalence. In this study 45% of the sample reported the engagement of moderate physical activity while only 9% were engaged in vigorous level of physical activity. Recent national survey indicated that only 17% of Saudi engaged in MVPA [41]. The observed difference of level of MVPA in our sample compared to the national prevalence might be due to several reasons. One main reason is the questionnaire used to collect physical activity data. In our study we used GPAQ that assess level of physical activity during the whole day where the questionnaire used on the Alqahtani et al. [41] focused on leisure time physical activity. Other reasons could be that our sample practice higher level of physical activity due to increase fear of COVID-19 and distress. A systematic review by Stults-Kolehmainen and Sinha concluded that in 18.2% of the reviewed prospective studies indicated that distress level was positively associated with physical activity [42]. Similar results in physical activity were observed in Lesser and Nienhuis [22] study that indicated Canadians reported higher physical activity level that is associated with distress level. This relation also was found in a study performed among adult twin pairs, where they illustrated that low level of physical activity associated with high level of perceived stress [43]. Another reason might be that participants in our study had more time to be physically activity because they are restricted to their home during lockdown period. Despite high levels of MVPA in our sample, the majority sample (60%) reported sitting for 6 hours or more per day. Prior to COVID-19 several studies reported that sedentary behavior levels in Saudi population ranged from 4.52 to 11.35 hours per day similar to our sample results [44–46].

Sleep assessment indicated that about half of our participants achieved the recommended sleep duration [47]. This prevalence was similar to previously reported levels in studies conducted prior to COVID-19 [48, 49]. However, about 58% of our sample reported poor sleep quality. This percentage is concerning as poor sleep quality is associated with increased risk of cardiovascular disease and diabetes [50, 51]. The results of the study highlight the need for widespread dissemination of recommendations of better sleep practices whether the participants had poor sleep quality before the start of the pandemic or not [52]. These recommendations include maintaining a regular sleep routine, practicing self-reflection, limiting exposure to COVID-19-related news or any distressful news, and engaging in regular exercise during daylight hours [52].

The early studies published during COVID-19 pandemic indicate that the pandemic has a negative effect on mental health [43, 53, 54]. However, the majority of the participants psychological distress results in this study indicated no to mild distress. These results were similar to studies that assessed psychological distress specifically during COVID-19 in Saudi population and other European countries [55–57]. These results is noteworthy as psychological distress might induces positive changes as seen in this study with physical activity or negative changes as seen with sleep and sedentary behavior. Further, this study reported fear of COVID-19 level to be low. However, a regression analysis indicated that participants with higher distress scores reported higher level of fear from COVID-19. Similar results were reported in studies from China, Australia and United States [58–60]. Thus, more depth investigation on the relationship of psychological distress and lifestyle behaviors are warranted [61].

Perceived social support has been shown to have a protective effect against depression and general health [62, 63]. Further, social isolation is associated with perceived social support and ultimately can impact mental health [64]. During COVID-19 times were social distancing is enforced about 10% of people in Switzerland felt socially isolated [65]. Majority of the participants (approximately 70%) in this study reported to have only minimal to moderate social support. This finding is of importance especially to primary health care physicians to increase their effort in screening for social isolation and depression in their patients.

The results of this study should be interpreted while taken the inherited limitations in account. The survey responses in this study were predominantly from Central region and female although the survey link was shared across all the region in Saudi Arabia through various social media platforms and was directed for both genders. This could be explained by the researchers’ use of snowball sampling technique. Therefore, findings of this study might be more generalizable to the central region and women rather than across Saudi Arabia population. Further, the snowball sampling method used may have introduced selection bias, where only those who received the study link and were internet-literate had the chance to participate. Considering the restrictions of movement and social distancing, an online survey was the only viable option during the pandemic to address our research objectives. Moreover, participants who completed all study survey sections response rate was low. This might be due to the combination of multiple surveys thus requiring a longer time commitment to answer these surveys. Nonetheless, this study provides invaluable information on the lifestyle behaviors during COVID-19 cautionary and emergency procedures.

In conclusion, the current study reported the prevalence of multiple lifestyle behaviors measured as a cluster and its association with fear of COVID-19 level. In the current sample, high level of sedentary behavior, poor sleep, and unhealthy dietary habits were prevalent. Further, being female and married were associated with high level of fear of COVID-19. From lifestyle behaviors only distress was associated with fear of COVID-19. Lifestyle behaviors are dynamic variables that can change overtime or with sudden changes in social or environmental norms such as COVID-19 pandemic. Thus, the scientific community can benefit from periodical assessments of lifestyle behaviors and their effect on health. Future research needs to establish the short-term and long-term effect of lifestyle behaviors as cluster on physical and mental health especially during these troubling times.

## Supporting information

S1 Data (XLSX)
